# Effects of *Sesamum indicum* L. Seed Extract on Male Reproductive Parameters and In Silico Anti‐Infertility Insights

**DOI:** 10.1155/adpp/9519972

**Published:** 2026-05-25

**Authors:** John I. Ogbu, Efe Omorodion-Osagie, Funmileyi O. Awobajo, Ibiyemi I. Olatunji-Bello

**Affiliations:** ^1^ Department of Physiology, Faculty of Basic Medical Sciences, College of Medicine, Lagos State University, Lagos, Nigeria, lasu.edu.ng; ^2^ Department of Physiology, Faculty of Basic Medical Sciences, College of Medicine, University of Lagos, Lagos, Nigeria, unilag.edu.ng; ^3^ Department of Physiology, Faculty of Biomedical Sciences, Kampala International University, Bushenyi, Uganda, kiu.ac.ug

**Keywords:** gene ontology, male infertility, network pharmacology, *Sesamum indicum* L., sperm analysis

## Abstract

The etiological contribution of male factors to infertility remains a significant concern in reproductive health. Natural dietary products are gaining attention as promising interventions to improve male infertility. This study investigates the effects of aqueous extract of *Sesamum indicum* L*. (S. indicum)* on male reproductive parameters and potential anti‐infertility mechanisms. Male Sprague–Dawley rats were randomly assigned to five groups: a control group (distilled water), two treatment groups receiving aqueous *S. indicum* extract (1.5 and 3.0 g/kg) for 30 days, and corresponding recovery groups. Reproductive organs were assessed for weight and sperm parameters. Blood serum was analyzed for luteinizing hormone (LH), testosterone, and lipid profile parameters. Incorporating in silico approaches, bioactive compounds of *S. indicum* were screened from a database, and their targets were predicted via network pharmacology, followed by functional enrichment analyses and molecular docking. Findings show that *S. indicum* significantly increased (*p* < 0.05) the weights of the hearts, adrenal glands, livers, and accessory reproductive organs. Sperm count and morphology, as well as LH and testosterone levels, were significantly increased (*p* < 0.05). Network pharmacology identified linoleic acid, oleic acid, biotin, and esculentic acid as key contributors to the extract’s anti‐infertility effects, potentially via interactions with EGFR, HIF1, BCL2, TNF, and AKT1. These interactions, supported by favorable binding affinities, may involve antioxidative pathways. Although the exposure duration, differences in extraction methods, paucity of experimental molecular data, and algorithmic constraints represent potential limitations, the present findings provide pharmacological insights into the therapeutic role of *S. indicum* in male infertility.

## 1. Introduction

Infertility is a growing global public health concern, with male factors accounting for approximately 50% of all cases [1, 2]. It is clinically defined as the failure to achieve pregnancy after at least 12 months of regular, unprotected sexual intercourse with a fertile partner [3]. Although studies on the syndrome have been conducted, there remains a paucity of robust data and a limited understanding of its underlying pathophysiology [4]. The etiology of male infertility is multifactorial, encompassing lifestyle and nutritional factors, medication use, environmental and occupational toxic exposures, endocrine dysfunction, and genetic abnormalities [2]. Clinically, it is commonly characterized by quantitative and qualitative sperm abnormalities, including reduced sperm concentration, impaired motility, abnormal morphology, or a combination of these defects [5].

Male fertility potential depends on the normal function of the testes and reproductive accessory organs, including sperm production, maintenance of good semen quality, and production of male sex hormones [6]. These indicators rely on cholesterol and other lipids, which are key players in spermatogenesis and the synthesis of male sex steroids [7–10]. Previous studies have shown that serum levels of triglycerides, high‐density lipoproteins (HDL), and low‐density lipoproteins (LDL) are linked with sperm morphology and semen quality [11]. Alterations in the normal metabolism of these lipids affect male reproductive parameters, including sperm motility, sperm count, sperm morphology, and circulating levels of male sex hormones [6].

Ongoing efforts to elucidate and restore male reproductive homeostasis have driven increasing interest in phytochemicals owing to their accessibility, affordability, and broad spectrum of biological activities. Natural dietary products are emerging as promising adjuncts to conventional infertility therapies, and systematic evaluation of their therapeutic potential may expand the range of options available to both clinicians and patients [2]. *Sesamum indicum* L. (*S. indicum*) is an herbaceous flowering plant from the Pedaliaceae family. Commonly referred to as the “queen of oilseeds,” it is rich in macronutrients and bioactive compounds with potential health benefits [12].

Several benefits of the seed have been documented: Sesame seed has been reported to have good oxidative stability and antioxidant, cardioprotective, anti‐inflammatory, and antitumor properties [13, 14]. Very few studies have investigated the reproductive effects of the seed in males amidst the current knowledge gap in understanding its underlying mechanisms [14]. Therefore, this study employed in vivo and in silico approaches to investigate the effects of aqueous *S. indicum* extract on male reproductive parameters and potential anti‐infertility mechanisms.

## 2. Materials and Methods

### 2.1. Preparation of Aqueous Extract of *S. indicum* Seed

*S*. *indicum* seeds used were purchased from a local grocery market in Mushin, Lagos State, during the dry season between October and November. Taxonomic identification of the plant was carried out by experts at the Department of Pharmacognosy herbarium at the University of Lagos. Identification and registration were carried out at the Forest Research Institute of Nigeria (FRIN), Ibadan. Voucher number FHI. 114515 was ascribed to the specimen deposit. After identification, the seeds were thoroughly washed with distilled water to remove adhering particles and then air‐dried. The dried seeds were ground using a blender and mixed with distilled water. The mixture was stirred thoroughly with a stirrer and then extracted using a Soxhlet extractor. The yield was then stored in an airtight bottle at ‐ 4°C until use. All procedures, including extract preparation and drug administration, as well as housing and euthanasia, were in accordance with the Helsinki guidelines for the care and use of experimental animals [15] as approved by the Institutional Health Research and Ethics Committee, College of Medicine, University of Lagos, Nigeria (Approval No. UNILAGACUREC/2025/03/AP09).

### 2.2. Animal Grouping and Treatments

Adult male Sprague–Dawley rats (average body weight of 144.70 ± 1.35 g) were used in these experiments. These animals were housed in spacious, well‐ventilated, and dry plastic‐and‐stainless‐steel cages with 5 rats per cage. They were fed a commercial pelleted rodent diet and water *ad libitum* at room temperature (26°C ± 2°C). The animals were kept in a controlled environment, in accordance with the standard operating procedures of the College of Medicine, University of Lagos, animal house facility. Animals’ weights were recorded weekly, and the average weight per group was calculated. The animals were divided into five groups, as shown in Table 1.

**TABLE 1:** Animal grouping and treatment.

Group	No of animal	Treatment	Duration (day)	Recovery (day)
Control	5	An equal volume of distilled water	30	—
1.5 g/kg treated	5	1.5 g/kg body weight/day of aqueous *Sesamum indicum* seed extract	30	—
3.0 g/kg treated	5	3.0 g/kg body weight/day of aqueous *S. indicum* seed extract	30	—
1.5 g/kg recovery	5	1.5 g/kg body weight/day of aqueous *S. indicum* seed extract	30	14
3.0 g/kg recovery	5	3.0 g/kg body weight/day of aqueous *S. indicum* seed extract	30	14

Each group received either distilled water or aqueous seed extract of *S. indicum* orally for 30 days. The control group received a volume of distilled water equivalent to that administered to the treatment groups. The 1.5 g/kg and 3.0 g/kg treated groups received aqueous *S. indicum* extract at 1.5 g/kg and 3.0 g/kg body weight, respectively. The corresponding 1.5 g/kg and 3.0 g/kg recovery groups were administered the same doses (1.5 and 3.0 g/kg, respectively) followed by a 14‐day recovery period. The selected doses were based on our previous acute oral toxicity study, which established its LD_50_ and safety profile [16]. The doses (1.5 and 3.0 g/kg body weight) used in this study were derived by diluting the LD_50_ to ensure safety while allowing evaluation of biological effects. The two recovery groups underwent 2 weeks of recovery after the initial 30 days of extract administration. The recovery groups were included to assess whether the effects of *S. indicum* seed extract on male reproductive parameters were reversible upon cessation of treatment.

This design enables the evaluation of physiological and biochemical recovery once exposure has ended. Five rats from each group, including the control, were sacrificed by cervical dislocation after CO_2_ asphyxiation. The recovery groups from the two treated groups were sacrificed at the end of the 2‐week recovery period. Blood samples were collected via cardiac puncture into heparinized sample bottles and used to prepare plasma for hormonal and lipid profile assays. The abdomen was dissected and opened, and the following organs were harvested over ice and weighed: liver, heart, adrenal gland, seminal vesicles, testis, and epididymis. Samples of these tissues were also stored in Bouin’s solution for histological studies. Samples from all experimental groups were processed using identical assays and assessments to ensure uniform handling.

### 2.3. Assessment of Sperm Parameters

Gross epididymal sperm motility was visually assessed under the microscope by an experienced researcher. Briefly, the epididymis was carefully dissected out and placed on a warm glass slide. It was carefully lacerated at the cauda part, and the white fluid, containing immotile sperm cells, was expressed onto the glass slide. A few drops of warm normal saline were added. The warm glass slide was transferred to a pre‐set light microscope stage and observed under a 40x objective. The gross motility score was used to assess sperm motility. The score was awarded between 100% for a rapidly forming cloud of motile sperm cells and 0% for a completely immotile sperm cell population. Care was taken to keep all instruments and fluids used in this study at body temperature to avoid thermal shock.

Sperm viability was evaluated using the eosin–nigrosin dye exclusion method, with live and dead sperm counted under a light microscope [17]. The caudal epididymis was carefully ground in a clean laboratory mortar and pestle. Epididymal sperm concentration was then determined with the help of the hemocytometer. Sperm count was expressed as a million/mL suspension. Sperm morphology was evaluated by counting sperm cells with abnormal morphology. Such abnormalities as a curved tail, a short tail, etc., were observed. The most common abnormality was a curved tail. The count was converted to a percentage. Morphology was reported as the percentage of normal sperm cells (total sperm cells in the fluid minus abnormal sperm cells, expressed as a percentage). Improvement was considered when the rate of normal sperm counts increased.

### 2.4. Serum Biochemical Assessment

Blood was obtained by cardiac puncture from the rats in each study group after anesthetizing them with ether. Each blood sample was collected in heparinized bottles, spun at 2500 rpm for 10 min in a desktop centrifuge at 10°C–25°C. Serum samples were assayed for luteinizing hormone (LH) and testosterone using the enzyme‐linked immunosorbent assay (EIA) technique. The rat ELISA assay kit (Elabscience) was used for the hormonal assay. Plasma concentrations of LDL cholesterol were determined with commercial kits (Randox Laboratories, Crumlin, England). HDL cholesterol was determined in plasma with the same commercial kits after LDL was precipitated with a heparin‐MnCl_2_ solution [18].

### 2.5. Network Pharmacology and Molecular Docking

#### 2.5.1. Screening for Intersecting Targets of *S*. *indicum* and Male Infertility

The bioactive compounds of *S. indicum* seeds were retrieved from the Indian Medicinal Plants, Phytochemical and Therapeutics (IMPPAT) database (https://www.cb.imsc.res.in/imppat/) [19]. The bioactive components were filtered based on established criteria: bioavailability (BA) and drug‐likeness. Compounds with OB ≥ 30% that met at least three of the Lipinski, Ghose, Veber, Egan, and Muegge criteria for DL were selected [20]. One compound that failed to meet the DL criteria was included because of its extensive biological activity [21, 22]. The potential targets of the bioactive compounds were identified using SwissTargetPrediction (http://www.swisstargetprediction.ch/index.php) and the SEA database (https://sea.bkslab.org/) and filtered for probability scores > 0 [23]. Male infertility‐related genes were scanned from the GeneCards database (https://www.genecards.org/) using the keyword “male infertility.” The intersection of targets between *S. indicum* and these genes was identified using Venny 2.1.0 [24].

#### 2.5.2. Protein–Protein Interaction (PPI) Network

The visual relationship among the intersecting targets was assessed using the STRING database for PPI analysis with a confidence score greater than 0.4 and a species limitation of “Human sapiens” [25]. The PPI analysis outputs were loaded into Cytoscape v3.10.3 (https://cytoscape.org/) software to analyze the compound–target network between *S. indicum* seeds and male infertility. The topological parameter of maximal clique centrality was applied to identify core targets. The top 10 targets were considered core key targets [20].

#### 2.5.3. Functional Enrichment and Pathway Analysis

The SR plot database (https://www.bioinformatics.com.cn/srplot) was used to perform and visualize enrichment analyses of Gene Ontology (GO) functional terms and Kyoto Encyclopedia of Genes and Genomes (KEGG) pathways for potential key targets in male infertility. GO terms—biological process, molecular function, and cellular component—were formed. Data were depicted as dot plots, sorted by the number of enriched targets, significance, and enrichment score [23, 25].

#### 2.5.4. Molecular Docking

Molecular docking was used to validate the binding affinity of bioactive compounds from S. *indicum* for core genes in the PPI network. Based on the descriptor values, the top four bioactive compounds and core target proteins were selected for molecular docking. The 3D structures of the bioactive compounds were obtained from PubChem and saved in PDB format. The Avogadro tool was used to add hydrogen bonds and optimize the compound. The 3D structures of the top five core genes, as identified through network pharmacology, were retrieved from the RCSB Protein Data Bank (PDB: https://www.rcsb.org) in 3D format, and their PDB IDs were recorded. X‐ray–solved crystal structures with higher resolution (up to 2.5 Å), derived from humans, served as the basis for selection. The protein structures were prepared using ChimeraX (v1.9) by removing cocrystallized ligands and nonessential residues. All water molecules were removed, and polar hydrogens and Gasteiger charges were added using AutoDock 4.2. This tool was then used to predict the binding affinities and interaction conformations of the *S. indicum* bioactive compounds with their respective targets. The resulting docked complexes were analyzed and visualized using Discovery Studio Visualizer to identify key amino acid interactions and binding orientations [26].

### 2.6. Statistical Analysis

The data obtained from this study were subjected to a one‐way analysis of variance (ANOVA) test using GraphPad Prism 8.0. Results are expressed as mean ± standard error of mean (SEM). The level of significance was placed at *p* < 0.05. Tables were used to present experimental results, while pie charts and dot plots were used to visualize network pharmacology findings.

## 3. Results

### 3.1. Effects of Aqueous Seed Extract of *S*. *indicum* on Mean Body and Organ Weights

The effects of *S. indicum* treatment on the mean body and organ weights are presented in Table 2 below. There was a significant decrease in body weight in rats treated with *S. indicum* 1.5 g/kg after 1 week of treatment (*p* < 0.05). In contrast, the group treated with *S. indicum* 3.0 g/kg recorded a significant increase (*p* < 0.05) in weight (203.83 ± 1.70 g) in the second week compared with the control group (191.20 ± 2.05 g). Both treated groups (1.5 and 3.0 g/kg) recorded a significant (*p* < 0.05) increase in weight compared with the control from the third week of extract administration onward, in the fourth week. The weight (203.83 ± 1.70 g) of the 3.0 g/kg group was significantly higher than that of rats administered *S. indicum* 1.5 g/kg (194.88 ± 1.4 g) from the second week of extract administration onward, the fourth and last week of the treatment. Both recovery groups (1.5 and 3.0 g/kg) showed significant increases in body weight compared with the control group.

**TABLE 2:** Effects of aqueous seed extract of *S*. *indicum* o*n* mean body weight of treated and recovery groups compared with control.

Week of treatment	Body weight (g)		
	Control	Extract administration	
		1.5 g/kg treated	3.0 g/kg treated
Week 0	143.21 ± 1.96	143.24 ± 1.93	146.64 ± 3.05
Week 1	182.27 ± 2.61	177.64 ± 1.46^∗^	182.28 ± 2.46
Week 2	191.2 ± 2.05	194.88 ± 1.41	203.83 ± 1.70^∗b^
Week 3	207.65 ± 1.40	212.43 ± 1.57^∗^	219.83 ± 2.30^∗b^
Week 4	234.82 ± 1.50	240.65 ± 1.93^∗^	253.25 ± 1.82^∗b^

	**Control**	**1.5 g/kg recovery**	**3.0 g/kg recovery**

Week 6	427.33 ± 4.58	436.24 ± 3.54^∗^	456.33 ± 1.72^∗b^

*Note:* 1.5 g/kg recovery = recovery group for rats treated with *S. indicum* 1.5 g/kg; 3.0 g/kg recovery = recovery group for rats treated with *S. indicum* 3.0 g/kg; *n* = 5.

^∗^ *p* < 0.05 (compared with control at the same week).

^b^ *p* <  0.05 (comparing both treated groups at the same week).

The effect of *S. indicum* seed on organ weight is presented in Table 3. Analysis of organ weights after administration showed that the testes, epididymis, liver, heart, and adrenal glands in the group treated with *S. indicum* 1.5 g/kg did not differ significantly from those in the control group. However, there was a significant increase (*p* < 0.05) in the weight of the seminal vesicle (0.46 ± 0.01 g) of the 1.5 g/kg treated group (0.43 ± 0.01 g) compared to the control. There was a significant increase (*p* < 0.05) in the weights of the testis (1.11 ± 0.01 g), epididymis (0.54 ± 0.02 g), liver (6.14 ± 0.01 g), and heart (0.57 ± 0.02 g) in the rats treated with *S. indicum* 3.0 g/kg compared to the control group. There was also a significant increase (*p* < 0.05) in the weight of the liver of the 3.0 g/kg treated group compared to that of the 1.5 g/kg.

**TABLE 3:** Effects of aqueous seed extract of *S*. *indicum* o*n* the reproductive organ weights in rats compared with the control and the recovery group.

Organ	Control	1.5 g/kg treated	3.0 g/kg treated	1.5 g/kg recovery	3.0 g/kg recovery
Testis (g)	1.05 ± 0.02	1.07 ± 0.02	1.11 ± 0.01^∗^	1.14 ± 0.03	1.16 ± 0.02
Epididymis (g)	0.47 ± 0.02	0.54 ± 0.02	0.54 ± 0.01^∗^	0.54 ± 0.02^∗^	0.55 ± 0.01^a^
Liver (g)	5.83 ± 0.03	5.90 ± 0.07	6.14 ± 0.01^∗b^	6.10 ± 0.07	6.37 ± 0.04^∗b^
Heart (g)	0.52 ± 0.01	0.55 ± 0.07	0.57 ± 0.02^∗^	0.71 ± 0.02	0.73 ± 0.02^∗^
Adrenal gland (g)	0.13 ± 0.01	0.14 ± 0.01	0.15 ± 0.01	0.22 ± 0.02	0.26 ± 0.01^∗^
Seminal vesicle (g)	0.43 ± 0.01	0.46 ± 0.01^∗^	0.46 ± 0.02	0.47 ± 0.02	0.49 ± 0.02^∗^

*Note:* 1.5 g/kg recovery = recovery group for rats treated with *S. indicum* 1.5 g/kg; 3.0 g/kg recovery = recovery group for rats treated with *S. indicum* 3.0 g/kg; *n* = 5.

^∗^ *p* < 0.05 (compared with control).

^a^ *p* < 0.05.

^b^ *p* < 0.05 (comparing both treated groups).

The *S. indicum* 3.0 g/kg recovery group had a significant increase (*p* < 0.05) in the weights of the epididymis (0.55 ± 0.01 g), liver (6.37 ± 0.04 g), heart (0.73 ± 0.02 g), adrenal gland (0.26 ± 0.01 g), and seminal vesicle (0.49 ± 0.02 g) when compared to the epididymis (0.47 ± 0.02 g), liver (5.83 ± 0.03 g), heart (0.52 ± 0.01 g), adrenal gland (0.13 ± 0.01 g), and seminal vesicle (0.43 ± 0.01 g) of the control group. There was also a significant increase (*p* < 0.05) in the weight (6.37 ± 0.04 g) of the liver of the 3.0 g/kg recovery group compared to the control (5.83 ± 0.03 g) (Table 3).

### 3.2. Effects of Aqueous Seed Extract of *S*. *indicum* on Sperm Parameters

The effects of *S. indicum* treatment on sperm parameters are presented in Table 4 below. These results for sperm parameters are shown in Table 4. The *S. indicum* 1.5 g/kg significantly increased (*p* ≤ 0.05) sperm count (48.56 ± 1.05 × 10^−6^ per mL) compared to the control (41.96 ± 2.25 × 10^−6^ per mL). The 1.5 g/kg recovery group recorded a significant increase in the sperm count (56.64 ± 2.66 × 10^−6^ per mL) compared to the control. There was a significant change (*p* < 0.05) in sperm morphology between the 1.5 g/kg (89.31 ± 2.92%) and the control group (78.47 ± 1.23%) following the administration of the extract. The sperm morphology of the higher‐dose (3.0 g/kg) group (82.52 ± 2.55%) also showed a significant increase (*p* < 0.05) compared to the control group (78.47 ± 1.23%). The recovery group showed no change in morphology after treatment. There was no significant difference in sperm cell viability between the treated groups and the control group.

**TABLE 4:** Effects of aqueous seed extract of *S*. *indicum* o*n* sperm parameters in the treated group compared with the control.

Reproductive parameters	Control	1.5 g/kg treated	3.0 g/kg treated	1.5 g/kg recovery	3.0 g/kg recovery
Sperm count (× 10^−6^ per mL)	41.96 ± 2.25	48.56 ± 1.05^∗^	34.34 ± 17.91	56.64 ± 2.66^∗b^	37.53 ± 5.28
Sperm motility (%)	66.22 ± 6.40	72.52 ± 3.50	72.71 ± 10.84	77.48 ± 1.91	88.24 ± 2.99
Morphology (%)	78.47 ± 1.23	89.31 ± 2.92^∗^	82.52 ± 2.55^∗b^	89.21 ± 3.67^∗^	82.43 ± 3.39
Viability (%)	40.42 ± 8.83	47.88 ± 7.07	55.36 ± 7.36	49.42 ± 3.82	58.14 ± 6.47

*Note:* 1.5 g/kg recovery = recovery group for rats treated with *S. indicum* 1.5 g/kg; 3.0 g/kg recovery = recovery group for rats treated with *S. indicum* 3.0 g/kg; *n* = 5.

^∗^ *p* < 0.05 (compared with control).

^b^ *p* < 0.05 (comparing both treated groups).

### 3.3. Effects of Aqueous Seed Extract of *S*. *indicum* on Blood Serum Lipid Profile and Hormones

There was a significant decrease (*p* < 0.05) in circulating HDL levels in rats treated with *S. indicum* 1.5 g/kg (42.08 ± 0.66 mg/dL) compared with the control group (55.38 ± 2.36 mg/dL). Similarly, the *S. indicum* 3.0 g/kg group also showed a significant decrease in HDL levels (*p* < 0.05) compared with the control group. The 1.5 g/kg recovery group 1 showed a significant decrease in circulating HDL levels (71.61 ± 1.61 mg/dL) compared with the control group (55.38 ± 2.36 mg/dL). Rats treated with *S. indicum* at 3.0 g/kg (39.16 ± 1.63 mg/dL) showed a significant decrease (*p* < 0.05) in circulating HDL levels compared with the control group (55.38 ± 2.36 mg/dL). The 3.0 g/kg recovery group (55.14 ± 1.20 mg/dL) showed a significant decrease (*p* < 0.05) in circulating HDL levels compared with the control (55.38 ± 2.36 mg/dL) and the 1.5 g/kg recovery group (71.61 ± 1.61 mg/dL).

The effect of the extract on circulating LDL levels was evaluated. There was a significant decrease in plasma LDL levels following treatment with *S. indicum* 1.5 g/kg (35.97 ± 0.82 mg/dL) compared with the control group (38.89 ± 0.28 mg/dL). This was, however, reversed, with a significant increase (*p* < 0.05) in circulating LDL levels in the *S. indicum* 3.0 g/kg recovery group (65.05 ± 1.87 mg/dL) compared to the control group (38.89 ± 0.28 mg/dL). There was also a significant decrease in plasma LDL levels in rats treated with *S. indicum* at 3.0 g/kg (33.68 ± 0.67 mg/dL) compared with the control group (38.89 ± 0.28 mg/dL). *S. indicum* 3.0 g/kg recovery group showed a significant decrease (*p* < 0.05) in the LDL levels (138.65 ± 1.28 mg/dL) compared with the control group (222.71 ± 2.51 U/L). In addition, there was a significant increase (*p* < 0.05) in LDL levels in rats administered *S. indicum* 3.0 g/kg (237.24 ± 0.75 mg/dL) compared with the control (222.71 ± 2.51 mg/dL) and the group administered *S. indicum* 1.5 g/kg (247.71 ± 2.49 mg/dL). However, plasma LDL levels were significantly lower in the *S. indicum* at 3.0 g/kg recovery group (141.50 ± 2.06 mg/dL) compared to the control (222.71 ± 2.51 mg/dL).

Hormonal levels in blood serum were assessed after administration of *S*. *indicum* extract. There was a significant difference in LH levels between the rats administered *S. indicum* 1.5 g/kg and the control group (Table 5). However, the *S. indicum* at 3.0 g/kg showed a significant increase (*p* < 0.05) in serum LH levels (15.23 ± 0.74 mlU/mL) compared to the control group (12.43 ± 0.93 mlU/mL). Serum testosterone levels were altered following administration of *S. indicum*. Although there was no significant change in the level of testosterone in the *S. indicum* 1.5 g/kg group after administering the extract, there was a significant decrease (*p* < 0.05) in the *S. indicum* 1.5 g/kg recovery group 1 (6.73 ± 0.27 ng/mL) compared to the control group (10.07 ± 0.58 ng/mL). On the contrary, there was a significant increase (*p* < 0.05) in the serum testosterone level in the rats administered *S. indicum* 3.0 g/kg (15.81 ± 0.73 ng/mL) compared to the control (10.07 ± 0.58 ng/mL) and 1.5 g/kg groups (12.27 ± 0.52 ng/mL).

**TABLE 5:** Effects of aqueous seed extract of *S. indicum* on blood serum lipid profile (HDL and LDL), lactate dehydrogenase (LDH), blood serum luteinizing hormone (LH), and testosterone level in the treated group compared with the control.

Group	Control	1.5 g/kg treated	3.0 g/kg treated	1.5 g/kg recovery	3.0 g/kg recovery
HDL (mg/dL)	55.38 ± 2.36	42.08 ± 0.66^∗^	39.16 ± 1.63^∗^	71.61 ± 1.61^∗^	55.14 ± 1.20^b^
LDL (mg/dL)	38.89 ± 0.28	35.97 ± 0.82^∗^	33.68 ± 0.67^∗^	65.05 ± 1.87^∗^	69.51 ± 2.04
LH (mlU/mL)	12.43 ± 0.93	13.22 ± 1.02	15.23 ± 0.74^∗^	14.40 ± 0.68	12.20 ± 0.74
Testosterone (ng/mL)	10.07 ± 0.58	12.27 ± 0.52	15.81 ± 0.73^∗^ ^b^	6.73 ± 0.27^∗^	9.70 ± 0.92

*Note:* 1.5 g/kg recovery = recovery group for rats treated with *S. indicum* 1.5 g/kg; 3.0 g/kg recovery = recovery group for rats treated with *S. indicum* 3.0 g/kg; *n* = 5.

^∗^ *p* < 0.05 (compared with control).

^b^ *p* < 0.05 (comparing both treated groups).

### 3.4. Network Analysis of Bioactive Compounds and Potential Targets of *S. indicum* in Male Infertility

**FIGURE 1:**
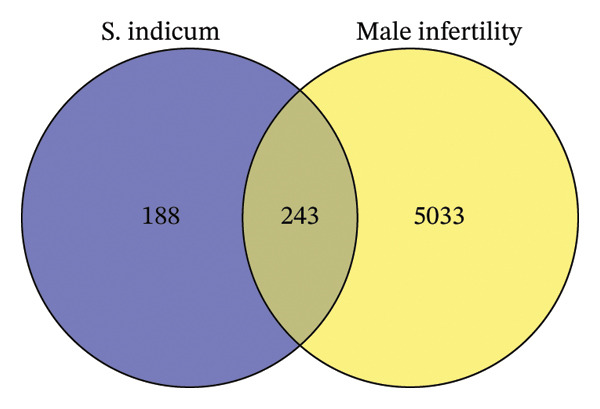
The Venn diagram showing the overlap of potential targets of *S. indicum* and male infertility‐related genes. There were 243 intersecting targets between the 188 *S. indicum* targets and 5033 male infertility‐related genes.

**FIGURE 2:**
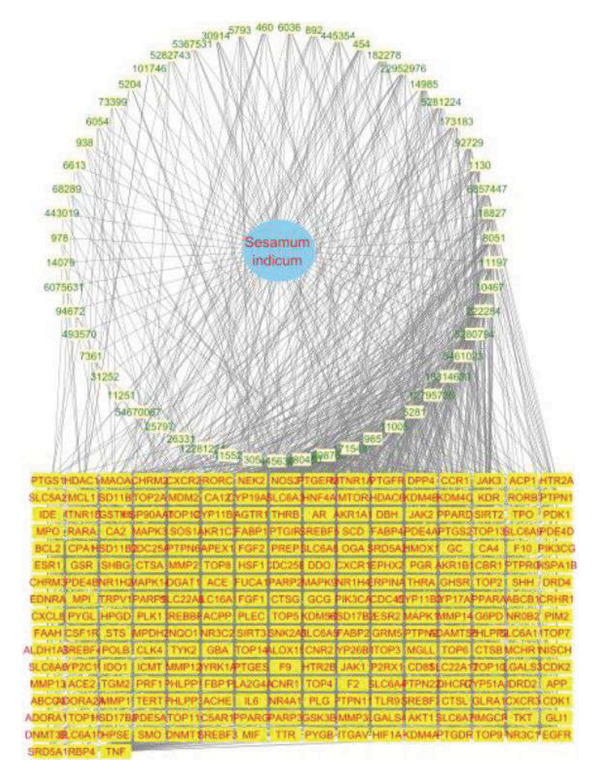
Potential S. *indicum*–compound–target network in male infertility. The central circular node represents *S. indicum*, surrounded by 55 bioactive compounds labeled by their PCID, while the grid layout represents their targets.

After screening of bioactive compounds of *S. indicum* based on the established criteria, a total of 55 bioactive compounds satisfied the criteria and were selected. Their PCID, BA, and DL are shown in Table 6. The SwissTarget and SEA databases identified 188 target proteins of *S. indicum* (Figure 1). By querying the GeneCards database for male infertility‐related genes, 5033 genes were found. Venny analysis identified an overlap of 243 genes. Following the query of GeneCards using “Male infertility” as the keyword, 5033 targets were found. We identified 250 overlapping targets that were considered intersecting (Figure 1). Cytoscape established an *S. indicum*–compound–target network (Figure 2). This network consists of 1 *S. indicum* node, 55 bioactive compound nodes, and 297 target nodes. The degree of the bioactive compounds was also analyzed in the *S. indicum*–compound–target network. The highest degree of bioactive compounds in *S. indicum* against male infertility depression were linoleic acid (58), oleic acid (58), biotin (51), and esculentic acid (50).

**TABLE 6:** Bioactive compounds of *S. indicum* seed.

Bioactive compounds	PCID	BA	DL
Esculentic acid	9898760	0.56	P
Thiamine	1130	0.55	P
3‐Methyl‐2‐butanone	11251	0.55	P
Myristic acid	11005	0.85	P
Tetracosanoic acid	11197	0.85	P
Octanal	454	0.55	P
Riboflavin	493570	0.55	P
Retinol	445354	0.55	P
2‐Acetylpyrrole	14079	0.55	P
3‐Methylbutanal	11552	0.55	P
1‐(3‐Methylfuran‐2‐yl)ethan‐1‐one	12281224	0.55	P
Sesamolin	101746	0.55	P
2‐Ethylpyrazine	26331	0.55	P
Acetylpyrazine	30914	0.55	P
Pinoresinol	73399	0.55	P
Choline	305	0.55	P
Stearic acid	5281	0.85	P
Astaxanthin	5281224	0.56	F
Sesamolinol	443019	0.55	P
1‐Octen‐3‐ol	18827	0.55	P
Hexadecenoic acid	5282743	0.85	P
Ascorbic acid	54670067	0.56	P
Cerotate	5641023	0.85	P
Furfuryl alcohol	7361	0.55	P
Sesamol	68289	0.55	P
Pantothenic acid	6613	0.56	P
Palmitic acid	985	0.85	P
Vitamin E	14985	0.55	P
Nicotinic acid	938	0.85	P
Pyrazine	9261	0.55	P
4‐Aminobenzoic acid	978	0.85	P
Inositol	892	0.55	P
delta7‐Avenasterol	12795736	0.55	P
Gamma‐tocopherol	92729	0.55	P
2,4‐Undecadienal	5367531	0.55	P
Arachidic acid	10467	0.85	P
Guaiacol	460	0.55	P
Beta‐tocopherol	6857447	0.55	P
4‐(5‐Methyl‐2‐furyl)‐3‐buten‐2‐one	6075631	0.55	P
Oleic acid	445639	0.85	P
D‐Galactose	6036	0.55	P
Campesterol	173183	0.55	P
Sesaminol	94672	0.55	P
1‐Methyldithio‐2‐propanone	22952976	0.55	P
Beta‐sitosterol	222284	0.55	P
Stigmasterol	5280794	0.55	P
5‐[3‐(1,3‐Benzodioxol‐5‐yl)‐1,3,3a,4,6,6a‐hexahydrofuro[3,4‐c]furan‐6‐yl]‐1,3‐benzodioxole	5204	0.55	P
Linoleic acid	5280450	0.55	P
2‐(4‐Methylpent‐3‐en‐1‐yl)‐1,4‐dihydroanthracene‐9,10‐dione	18314639	0.55	P
2‐Heptanone	8051	0.55	P
Kobusin	182278	0.55	P
D‐Glucose	5793	0.55	P
2,5‐Dimethylpyrazine	31252	0.55	P
2,5‐Diethylpyrazine	25797	0.55	P
Biotin	171548	0.56	P

*Note:* PCID, PubChem compound identification; BA, bioavailability score.

Abbreviation: DL, drug‐likeness.

### 3.5. PPI Network of the Intersecting Genes

Figure 3(a) shows the visual output of the PPI network construction of the 243 intersecting genes by STRING data. The hub targets of *S. indicum* against male infertility were identified as EGFR, HIF1, BCL2, TNF, AKT1, ESR1, IL6, MTOR, HSP90AA, and PPARG (Figure 3(b)).

FIGURE 3PPI interaction network of the intersecting targets. (a) The nodes represent the intersecting targets of *S. indicum* and male infertility, and the edges represent interactions between proteins. (b) The interaction among the 10 hub targets of the PPI network.

**Figure:**
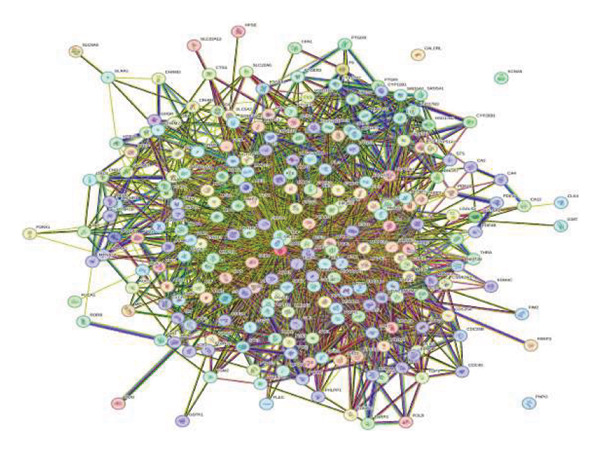
(a)

**Figure:**
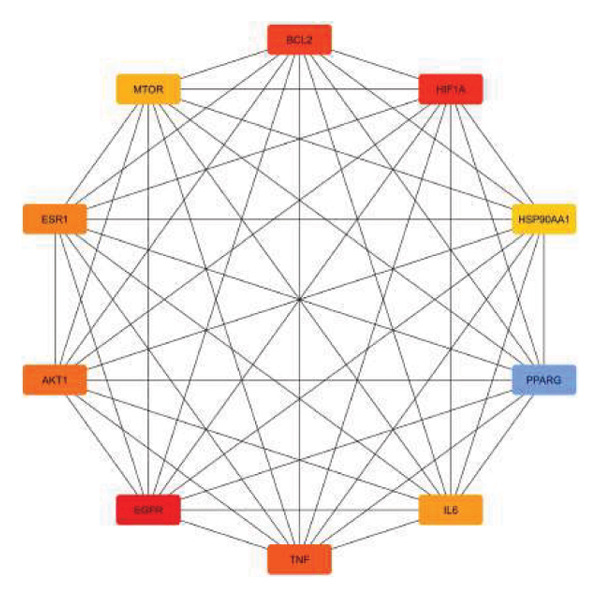
(b)

### 3.6. GO and KEGG Pathway Analyses

After filtering with *p*‐value < 0.01 and a minimum of 3 GO entries, analyses revealed 2138 GO entries and 124 KEGG enrichment analyses. There were 1906 biological processes, 59 cellular components, and 173 molecular functions, accounting for the total GO entries. The top 10 GO and KEGG enrichment findings are shown in dot plots in Figure 4.

FIGURE 4Gene Ontology (GO) and Kyoto Encyclopedia of Genes and Genomes (KEGG) enrichment analyses of 243 potential targets for S. indicum against male infertility. (a). Top 10 GO‐molecular function terms. (b) Top 10 GO‐biological process terms. (c) Top 10 GO‐cellular component terms. (d) Top 10 KEGG pathway.

**Figure:**
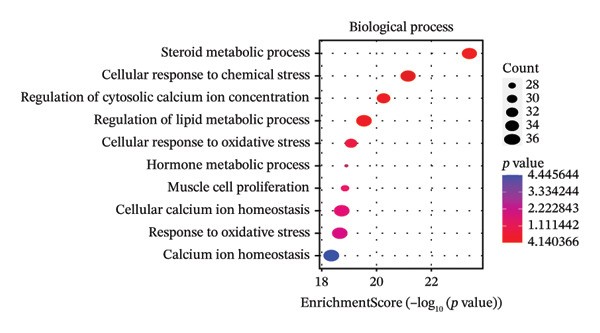
(a)

**Figure:**
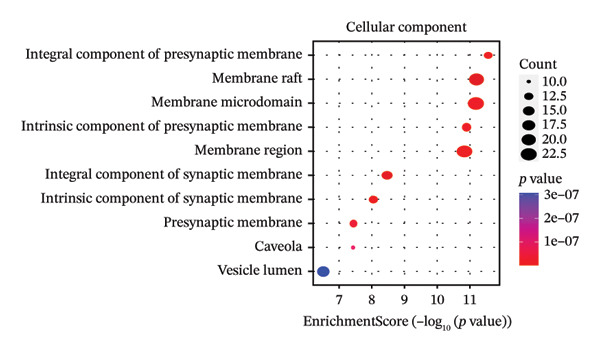
(b)

**Figure:**
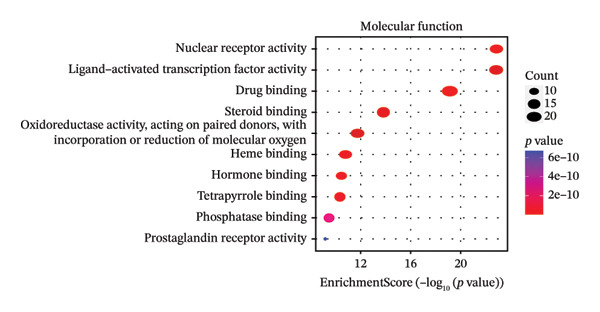
(c)

**Figure:**
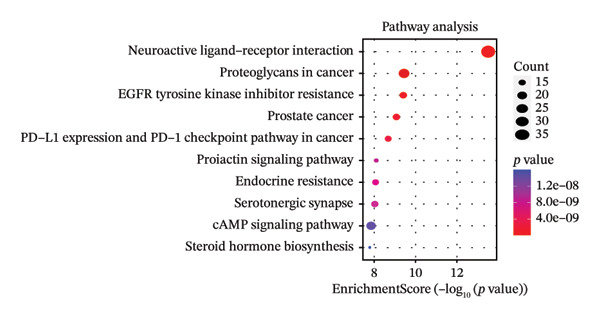
(d)

### 3.7. Molecular Docking

To further explore the binding activity of *S. indicum* to its target proteins, we selected the top 5 core hub genes (EGFR, HIF1, BCL2, TNF, and AKT1) and their corresponding PDB IDs for molecular docking. As shown in Table 7, the docking results demonstrated favorable binding of the top four *S. indicum* bioactive compounds (linoleic acid, esculentic acid, oleic acid, and biotin) to the selected core genes. The binding energies ranged from −3.87 to −10.02 kcal/mol. Esculentic acid demonstrated the highest binding affinity toward all selected targets, with binding energies of −10.02 kcal/mol (EGFR), −7.40 kcal/mol (HIF1), −7.19 kcal/mol (BCL2), −8.84 kcal/mol (TNF), and −7.78 kcal/mol (AKT1). The four bioactive compounds all demonstrated higher binding affinities for TNF than adalimumab (−6.53 kcal/mol), the protein’s reference compound. Figure 5 shows the molecular docking poses of the ligands in their best‐binding interactions with the key targets.

**TABLE 7:** Binding energy (kcal/mol) of the top 4 bioactive compounds of *S. indicum* and selected core genes.

Bioactive compounds (with their PCID)	EGFR (6tfv)	HIF1 (3hqr)	BCL2 (1ysw)	TNF (7jra)	AKT1 (3cqu)
Linoleic acid (5289450)	−7.13	−4.33	−4.75	−7.61	−7.11
Oleic acid (445639)	−5.47	−3.87	−4.34	−7.30	−6.48
Biotin (171548)	−6.71	−5.12	−5.79	−7.32	−6.72
Esculentic acid (9898760)	−10.02	−7.40	−7.19	−8.84	−7.78
Reference compound	−8.77^a^	−5.92^b^	−10.33^c^	−6.53^d^	−10.77^e^

^a^Osimertinib (PCID:71496458).

^b^7‐Hydroxyneolamellarin A (PCID:24179494).

^c^Venetoclax (PCID: 49846579).

^d^Adalimumab (PCID:107706).

^e^IQO (PCID:135398501).

FIGURE 5Molecular docking results of some ligand–target interactions in 3D (right side) and 2D (left side) images for (a) linoleic acid and EGFR, (b) linoleic acid and TNF, (c) biotin and TNF, and (d) esculentic acid and AKT1.

**Figure:**
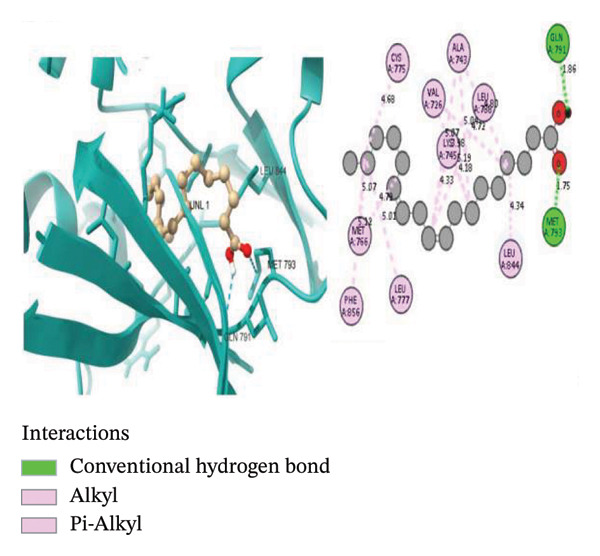
(a)

**Figure:**
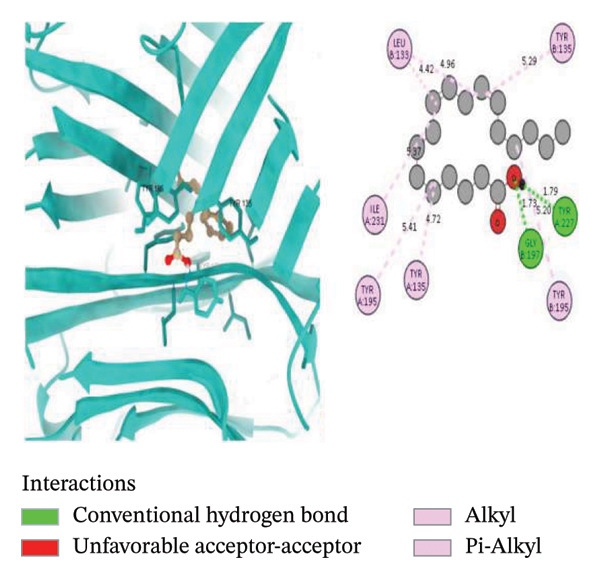
(b)

**Figure:**
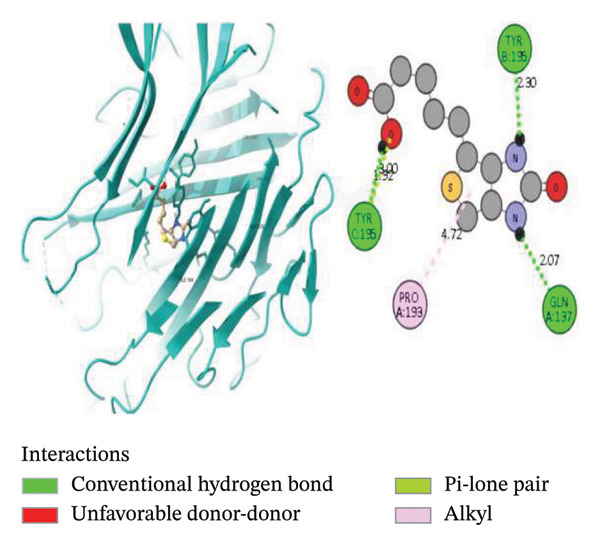
(c)

**Figure:**
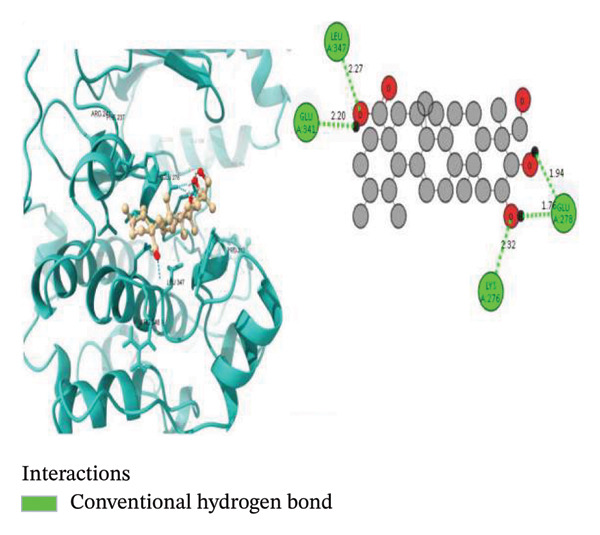
(d)

## 4. Discussion

Natural products are gaining interest as adjuncts in managing male infertility, based on accumulating evidence of a potential role in modulating reproductive functions [2, 6]. *S. indicum*, a nutritionally rich seed with health‐promoting benefits, remains insufficiently characterized in this context. Here, we investigated the effects of a 30‐day treatment with the aqueous seed extract of *S. indicum* on reproductive indices and the serum lipid profile in rats. Network pharmacology and molecular docking were integrated to elucidate potential mechanisms underlying the extract’s effect. Results indicate that the extract increases body and organ weights and sperm indices, while modulating male sex hormone levels and the lipid profile in rats. Network pharmacology identified EGFR, HIF1, BCL2, TNF, and AKT1, among others, as key targets of *S. indicum*, with favorable binding affinities for its bioactive compounds.

*S*. *indicum* promotes growth [27]. The observed weight gain, consistent with previous studies, suggests anabolic effects and may be attributed to the seed’s high caloric content and abundance of unsaturated fatty acids [28, 29]. Although contrasting findings on this effect exist, they may be attributed to seed processing or transformation. For example, sesamin, sesamol, and sesamolin are compounds derived from processed and denatured seeds of *S. indicum*. These lignan products have been reported to reduce body weight [10, 30]. The weights of animals at 6 weeks were roughly double those at 4 weeks, while the organ weights did not follow the same trend. The observed increase in body weight, particularly in the recovery group at week 6, may be attributed to enhanced adipose deposition rather than a proportional increase in organ mass. It is important to note that total body weight reflects the combined mass of organs and surrounding fat, whereas organ weights were determined after careful excision and removal of adherent adipose tissue [31]. This distinction may explain the apparent discrepancy between whole body and organ weight changes.

Sperm cell parameters, including count, morphology, and viability, are critical in male reproductive function [1]. In this study, the *S. indicum* 1.5 g/kg significantly increased sperm count compared with the control group. The marked increase in sperm count observed in the 3.0 g kg^−1^‐dose recovery group likely reflects enhanced spermatogenesis during the post‐treatment period. Collectively, these changes indicate that the 30‐day treatment, corresponding to approximately half of a full spermatogenic cycle, may prime the testes for optimal spermatogenic activity. Sperm morphology was also significantly improved, although motility was not markedly altered. These findings are consistent with previous reports on the spermatogenic effects of *S. indicum* [14, 27, 29], suggesting that the extract enhances both spermatozoa quantity and structural integrity, key surrogates of spermatogenic efficiency [5]. Sperm viability between groups did not differ significantly. The recovery groups showed a numerical increase in sperm viability relative to the control and their respective treatment groups, suggesting a recovery trend following treatment withdrawal. Increased testicular, epididymal, and seminal vesicle weights further support enhanced reproductive activity [32].

Lipid homeostasis significantly influences male fertility [11]. The relationship between lipids and human fertility is plausible, as cholesterol plays a key role in the synthesis of sex steroid hormones and is a determinant of sperm production [9, 10]. *S*. *indicum* recorded a significant reduction in the levels of serum lipids (HDL and LDL) in both treated groups compared to the control group. The reduction in both serum HDL and LDL levels following administration of the extract contrasts with the typical lipid‐modulating effects reported for *S. indicum* in normal conditions, which generally include decreased LDL and preserved or elevated HDL [33]. Although few studies have reported lipid‐lowering effects, these were primarily under hyperlipidemic conditions [34, 35]. This atypical pattern reflects alterations in lipid metabolism rather than overt toxicity, given that no weight loss or organ damage was observed. Additionally, the use of an aqueous extract, which differs in phytochemical composition from lipid‐rich preparations, may account for these discrepancies [27]. Importantly, the recovery of lipid and reproductive parameters post‐treatment suggests a reversible metabolic adaptation. While reduced lipid availability could theoretically impact steroidogenesis, the increase in sperm count, morphology, and hormonal levels suggests involvement of alternative mechanisms yet to be understood.

Male fertility relies on adequate testosterone levels, which are crucial for the structural and functional integrity of spermatozoa, the testes, and other androgen‐dependent accessory organs [36]. In this study, serum testosterone levels were significantly higher in the 3.0 g/kg than the 1.5 g/kg and control groups, corroborating earlier findings linking *S. indicum* treatment to increased androgen production [27, 29]. The hormonal elevation may be accounted for by increased endocrine testicular activity, as reflected by increased testicular size, an important indicator of androgenic stimulation and a predictor of male reproductive capacity [32]. The elevation in LH levels may be attributed to the stimulation of the anterior pituitary within the hypothalamic–pituitary–gonadal axis [5, 6]. Such stimulation enhances endogenous testosterone production, thereby supporting a hormonal environment conducive to optimal spermatogenesis. Importantly, the possibility of a direct effect of the extract on Leydig cells cannot be excluded, as some bioactive compounds act locally to potentiate steroidogenesis independently of pituitary mediation [37].

Mechanisms linking *S. indicum* to its reproductive effects are poorly understood. In silico approaches, including network pharmacology and molecular docking, offer cost‐effective and time‐efficient strategies for predicting molecular targets and accelerating drug discovery [25]. Network analysis suggests that bioactive compounds of *S. indicum* interact with multiple molecular targets, which may underlie its effects on male reproductive parameters. Core targets, including EGFR, HIF1, BCL2, TNF, and AKT1, which have been implicated in male infertility, underscore the significance of our network pharmacology analysis. Their dysregulation constitutes molecular culprits that lead to spermatogenic and sperm maturation arrest and oxidative stress in male infertility. EGFR governs cell proliferation, survival, and differentiation. Dysregulated EGFR overexpression is linked to male sterility [38], while its modulation by *S. indicum* compounds may enhance testicular growth and sperm output. HIF1 participates in hypoxic adaptation by regulating angiogenesis, metabolism, and redox balance; its dysregulation disrupts testicular homeostasis, driving oxidative stress and impaired spermatogenesis [39]. BCL2, a key anti‐apoptotic regulator of mitochondrial pathways, preserves germ‐cell survival during spermatogenesis; its downregulation is associated with male infertility [40]. Upregulation or stabilization of BCL2 signaling by *S. indicum* may improve sperm cell parameters by inhibiting germ‐cell apoptosis.

Moreover, TNF is a pleiotropic cytokine that controls inflammation, the balance between cell death/survival, and immune responses. Although the mechanisms remain unclear, the pro‐inflammatory cytokine has been implicated in sperm dysfunction. Chronic elevation impairs sperm motility and induces oxidative stress [41]. The TNF indication in the network analysis suggests that *S. indicum*’s effects on reproductive parameters may involve modulation of inflammatory pathways. AKT1 is crucial in testicular somatic and germ cells for regulating spermatogonial proliferation, differentiation, and Leydig cell steroidogenesis [42]. Activation of AKT1 pathways may contribute to the extract’s effects on testicular growth, supporting testosterone production and enhancing spermatogenesis. Additional targets, including ESR1 and mTOR, further support involvement of endocrine and growth‐regulatory pathways.

GO​ enrichment analysis highlighted key biological themes underlying the potential target–pathway networks through which *S. indicum* may exert its effects in male infertility. Enrichment of genes within the biological process category suggests that steroid metabolism, hormone regulation, oxidative stress responses, rhythmic processes, and cytosolic Ca^2+^ regulation may play a critical role in the extract’s effects. These metabolic processes may explain the significant changes in serum lipid and hormonal levels observed with the seed extract. As revealed in the molecular function category, molecular activities driving these biological processes may primarily involve nuclear receptor activity, ligand‐activated transcription factor activity, steroid binding, oxidoreductase activity, etc. [38]. Concurrently, KEGG enrichment analysis implicates oxidative stress–related pathways as potential mediators of the extract’s effects in the male reproductive system [13]. In addition, the identified cellular components, mainly membrane structures, from our GO enrichment analysis are consistent with previous studies [43]. The KEGG‐enriched pathways provide a plausible mechanistic basis for the seed’s reproductive effects.

Molecular docking results showed binding affinities ranging from −10.02 to −3.87 kcal/mol, indicating potential interactions requiring experimental validation. The stability of ligand–target binding depends on the binding energy. The lower the binding energy of the complex, the more stable the binding interaction. Binding affinity values < 0 kcal/mol indicate that the ligand can bind to the receptor; values ≤ −5.13 kcal/mol indicate stronger binding [23]. Among the screened bioactive compounds, esculentic acid exhibited the highest binding affinity toward the five core target genes. However, its affinity was lower than that of the reference inhibitors for BCL2 (venetoclax) and AKT1 (IQO). The strong docking performance of this pentacyclic triterpenoid is likely due to its ability to establish multiple stabilizing interactions, including hydrogen bonds and hydrophobic contacts, within the target proteins’ active sites [44]. These in silico findings support the potential role of esculentic acid in *S. indicum*’*s* effect. Linoleic acid and biotin, compounds previously associated with improved semen quality and sperm survival, demonstrated high binding affinities for EGFR, TNF, and AKT1 [45, 46]. Considering the role of these targets in growth, inflammatory, and steroidogenic signaling pathways that regulate spermatogenesis, the hypothalamic–pituitary–gonadal axis, and sperm maturation [38, 41], the docking results suggest a plausible mechanistic basis that warrants further experimental validation.

Several limitations of our study exist, highlighting important directions for future research. The 30‐day exposure does not encompass the full spermatogenic cycle, which requires about 60 days, limiting conclusions on long‐term reproductive effects [47]. The absence of histological and experimental molecular validation, reliance on database‐driven predictions, and limited sample size further constrain interpretation. Future studies incorporating extended treatment durations, mechanistic assays, and standardized extract characterization are warranted to validate these observations and clarify underlying pathways.

## 5. Conclusion

Altogether, the aqueous seed extract of *S. indicum* significantly increased sperm count and reproductive organ indices, accompanied by changes in serum hormonal and lipid profiles. Integrative network pharmacology and molecular docking suggest that these effects may be potentially mediated by favorable interactions between its bioactive compounds and core targets, including EGFR, HIF1, BCL2, TNF, and AKT1. These interactions may activate steroidogenesis, oxidative stress, inflammation, and cell survival pathways. Collectively, these findings support the potential of *S. indicum* as a nutraceutical candidate for managing male infertility. However, further studies incorporating longer treatment durations and experimental validations of identified targets are required to elucidate the therapeutic relevance of *S. indicum* in male infertility.

**Nomenclature**
AKT1: Protein kinase B isoform 1
BCL2: B‐cell lymphoma 2 (BCL2)
EGFR: epidermal growth factor receptor
ESR1: estrogen receptor 1
HIF1: hypoxia‐inducible factor 1
HSP90AA1: heat shock protein 90 alpha family class A member 1
mTOR: mammalian target of rapamycin
PDB ID: Protein Data Bank identifier
PI3K: phosphoinositide 3‐kinase
TNF: tumor necrosis factor

## Author Contributions

Ibiyemi I. Olatunji‐Bello and Funmileyi O. Awobajo conceptualized, designed the experiment, and provided an original draft of the manuscript. Efe Omorodion‐Osagie and John I. Ogbu were involved in the experimentation and in the writing and revision of the manuscript.

## Funding

No funding was received for this research.

## Conflicts of Interest

The authors declare no conflicts of interest.

## Data Availability

The data used to support the findings of this study are available from the corresponding author upon reasonable request.
